# Artificial Neural Network for Discrimination and Classification of Tropical Soybean Genotypes of Different Relative Maturity Groups

**DOI:** 10.3389/fpls.2022.814046

**Published:** 2022-07-12

**Authors:** Lígia de Oliveira Amaral, Glauco Vieira Miranda, Bruno Henrique Pedroso Val, Alice Pereira Silva, Alyce Carla Rodrigues Moitinho, Sandra Helena Unêda-Trevisoli

**Affiliations:** ^1^Laboratory of Biotechnology and Plant Breending, Department of Agricultural Sciences, São Paulo State University - UNESP/FCAV, Jaboticabal, Brazil; ^2^Department of Agronomy Coordination, Federal Technological University of Paraná, Curitiba, Brazil

**Keywords:** machine learning, photoperiod, *glycine max*, relative maturity, data mining, apparent error rate

## Abstract

Soybean has a recognized narrow genetic base that often makes it difficult to visualize available genetic and phenotypic variability and identify superior genotypes during the selection process. However, the phenotypic expression of soybean plants is highly affected by photoperiod and the cultivation of a given variety is performed in the latitude range that presents ideal conditions for its development based on its relative maturity group (RMG) for the optimization of the phenotypic expression of its genotype. Based on the above, this study aimed to evaluate the efficiency of artificial neural networks (ANNs) as a tool for the correct discrimination and classification of tropical soybean genotypes according to their relative maturity group during the population selection process with the aim of optimizing the phenotypic performance of these selected genotypes. For this purpose, three biparental populations were synthesized, one with a wide genetic variability for the RMG character obtained from the hybridization between genitors of maturity groups RMG 5 (Sub-tropical 23° LS) × RMG 9.4 (Tropical 0° LS) and two populations with a narrow variability obtained between genitors RMG 7.3 (Tropical 20° LS) × RMG 9.4 and RMG 5.3 × RMG 6.7, respectively. Criteria for comparing the developed ANN architecture with Fisher’s linear and Anderson’s quadratic parametric discriminant methodologies were applied to the data for the discrimination and classification of the genotypes. ANN showed an apparent error rate of less than 8.16% as well as a low influence of environmental factors, correctly classifying the genotypes in the populations even in cases of reduced genetic variability such as in the RMG 5 × RMG 6 population. In contrast, the discriminant functions were inefficient in correctly classifying the genotypes in the populations with genealogical similarity (RMG 5 × RMG 6) and wide genetic variability, with an error rate of more than 50%. Based on the results of this study, ANN can be used for the discrimination of genotypes in the initial generations of selection in breeding programs for the development of high performance cultivars for wide and reduced photoperiod amplitudes, even with fewer selection environments, more efficiently, and with fewer time and resources applied. As a result of similarity between the parents, ANN can correctly classify genotypes from populations with a narrow genetic base, in addition to pure lines and genotypes with a high degree of inbreeding.

## Introduction

Soybean is the most important oilseed in the world and whose genetic improvement plays a significant role in the continuous growth of the crop. Soybean is highly sensitive to the photoperiod and considered a short-day species. The day length at the location of cultivation directly affects plant growth. In addition, the photoperiod influences the change from vegetative to reproductive stage, and consequently, the flowering, total cycle, and grain yield (Garner and Allard, 1930), consequently influencing the entire phenotypic performance in the field. Owing to this influence, the optimum photoperiod conditions for cultivars are restricted to a certain latitude range according to the environment in which the genotypes are cultivated (Lin et al., 2021). In Brazil, cultivars are distributed among 13 relative maturity groups (RMGs) classified geographically based on plant growth and development. Owing to the large territorial extent and latitude variation, Brazil comprises RMGs 5–9, respectively, from the south (latitude 30°) to the north of the country (latitude 0°) (Alliprandini et al., 2009).

The cultivation of a variety in an RMG different from its suitable one may result in undesired cycle elongation or reduction, insufficient or exaggerated vegetative development, susceptibility to pests and diseases specific to a certain time of the year, and low productivity (Miladinović and Đorđević, 2011), impairing their phenotypic performance. Genetically, the time to reach flowering and maturity is controlled by genes *E*. Thus far, 11 major *loci* (*E1–E11* and *J*) involved in the control of these characteristics have been identified in soybean (Samanfar et al., 2017). In general, except for *E6*, *E9*, *E11,* and *J* genes, the dominant allele of *E* genes leads to late flowering and maturity, whereas an increase in the number of recessive alleles entails precociousness of the variety (Lin et al., 2021).

The yield potential of cultivars at their appropriate production locations is maximized. Correct estimation of the phenological stages of the soybean plant allows improvement in the flexibility to modify its development as a whole and use the characteristics controlled by other genes that are affected by the durations of the vegetative and reproductive stages. The number of nodes and pods, growth habit, and characteristics related to the occurrence of higher temperatures at a certain stage of the plant such as oil and protein content and nitrogen and phosphor concentrations in grains, can be explored well, depending on the objectives of the breeding program (Miladinović et al., 2018).

The effect of a phenotype is the sum of the genetic effects, environmental effects, and their interaction. The evolution of technology and the improvement of methodologies in breeding programs aim to isolate the environmental effects to the maximum extent to increase the efficiency of the selection of genotypes based on the genetic effects. Artificial neural networks (ANNs) have a high capacity in predicting, recognizing, discriminating, and classifying patterns. Moreover, different from parametric statistical approaches, they capture the complex characteristics of a dataset in addition to being slightly susceptible to noise and outliers and being suitable for nonlinearly separable problems common to agricultural experimentation (Kavzoglu and Mather, 2003; Sudheer et al., 2003; Haykin, 2008). Currently, at the experimental level, ANN models have been used in the prediction of genetic values (Soares et al., 2015), adaptability and stability (do Carmo Oda et al., 2019), phenotyping (Sá, 2018), yield estimates (Lu et al., 2022), genetic diversity (Rahimi et al., 2019; Taratuhin et al., 2020), disease detection, and classification (Hang et al., 2019; Trivedi et al., 2021). Moreover, they have demonstrated that the efficiency in the breeding stages can be increased, which can reduce the time and cost of obtaining high-performance cultivars.

Based on the above, the objective of the present study was to evaluate the efficiency of ANNs as a tool for the correct discrimination and classification of tropical soybean genotypes according to their relative maturity group during the population selection process, with the aim of optimization of the phenotypic performance of these selected genotypes. The possible application of this analysis is expected to obtain high performance cultivars for a wide range of photoperiods in soybean growing regions in Brazil.

## Materials and Methods

### Plant Material

Three soybean populations with different ranges of genetic variability were synthesized and evaluated for their RMG characteristics. Hybridizations were performed at the Department of Agricultural Production Sciences, Faculty of Agricultural Sciences, São Paulo University - UNESP/FCAV located at latitude 21°14′58″S and longitude 48°17′08″W, in Jaboticabal, São Paulo, Brazil. The population with a wide genetic variability for the RMG character was obtained from the hybridization between the genitors BMX Veloz (RMG 5.0; sub-tropical 23° LS) × BRS 278 RR (RMG 9.4; tropical 0° LS), called the Brazil population. The Northern and Southern populations, characterized by the most restricted variability for the character group of relative maturity, were established from crosses between the cultivars BRS 245 RR (RMG 7.3; Tropical 20° LS) and BRS 278 RR (RMG 9.4) and between cultivars BMX Energia (RMG 5.3) and BMX Potência (RMG 6.7), respectively. The controls of each population were their respective genitors, in addition to the cultivars TMG 7262 RR (RMG 6.2), TMG 1174 RR (RMG 7.4), and TMG 1179 RR (RMG 7.9).

The parent cultivar BRS 278 RR was approximately 73 cm tall, had an average cycle of 115–127 days, determined growth habit, brown pubescence, purple flower color, RMG of 9.4, and resistance to lodging, *Xanthomonas axonopodis*, *Cercospora sojina*, and *Diaporthe phaseolorum*, were susceptible to common soybean mosaic, cyst nematode, *Meloidogyne incognita,* and *Meloidogyne javanica*, and tolerant to stem necrosis virus (CpMMV). BMX Veloz was of medium size, had an average cycle of 120 days, indeterminate growth habit, light brown pubescence color, purple flower color, RMG of 5.0, was resistant to *D. phaseolorum* and *Phytophthora sojae* (*RPS1k* gene), and was moderately resistant to *C. sojina* and *X. axonopodis*. BMX Energia was of medium size, had an indeterminate growth habit, gray pubescence color, purple flower color, RMG of 5.3, resistance to *D. phaseolorum*, and moderately resistant to *C. sojina* and *X. axonopodis*. BMX Potência had a height of 90 to 100 cm, indeterminate growth habit, gray pubescence color, white flower color, RMG of 6.7, was susceptible to the nematode *M. incognita* and moderately resistant to the nematode *Meloidogyne javanica*, and resistant to lodging, *D. phaseolorum*, *C. sojina,* and *Phytophthora sojae*. BRS 245 RR had a height of 70 to 94 cm depending on the planting region, determined growth habit, brown pubescence color, white flower color, RMG of 7.3, was resistant to *D. phaseolorum*, *C. sojina,* and common soybean mosaic, tolerant to stem necrosis virus, moderately susceptible to powdery mildew, and susceptible to cyst nematodes, *M. incognita,* and *M. javanica*.

All genitor cultivars were transgenic, with resistance to the herbicide glyphosate.

To perform the analyses, the controls were considered as distinct populations and there were 11 populations in total (Table 1).

**Table 1:** Genealogy and relative maturity group (RMG) of 11 soybean populations used in study.

Population	Genealogy	RMG
Brazil	BRS 278 RR × 5953 RSF RR	9.4/5.0
Southern	BMX Potência RR × BMX Energia RR	6.7/5.3
Northern	BRS 245 RR × BRS 278 RR	7.3/9.4
GBN1	BRS 278 RR	9.4
GB2	5953 RSF RR	5.0
GS1	BMX Potência RR	6.7
GS2	BMX Energia RR	5.3
GN2	BRS 245 RR	7.3
TGM7	TMG 1174 RR	7.4
TGM6	TMG 7262 RR	6.2
TGM8	TMG 1179 RR	7.9

### Experimentation

Jaboticabal is located at latitude 21°15′19″S, presenting ideal photoperiod conditions for RMG genotypes 6–8 originating from the long rainy period in the region from November (spring) to April (autumn), allowing the cultivation of soybean cultivars with a cycle of up to 150 days. Field evaluation data were obtained from years 2017/2018, 2018/2019, and 2019/2020, corresponding to the filial generations from F_3_ to F_6_ for the Brazil population and F_4_ to F_7_ for the Northern and Southern populations. The three populations were conducted in the three agricultural years with a variable number of progenies, in addition to four commercial cultivars as controls within each population. The Brazil population consisted of 220 progenies in 2017/2018, 252 in 2018/2019 and 252 in 2019/2020. The Southern population consisted of 120 progenies in 2017/2018, 168 in 2018/2019 and 168 in 2019/2020. The Northern population consisted of 60 progenies in 2017/2018, 60 in 2018/2019 and 104 in 2019/2020. The agronomic characteristics evaluated were: number of days to flowering (NDF), number of days to maturity (NDM), total crop cycle (CYCLE), first pod insertion height (AIV), plant height at maturity (APM), lodging (Ac), agronomic value (VA), and grain yield (PG). Ac was evaluated based on a visual rating scale ranging from 1 (all plants erect) to 5 (all plants lodged), and VA was evaluated with a visual grading scale ranging from 1 (plants with poor agronomic characteristics) to 5 (plants with excellent agronomic characteristics). All experiments (three populations in three crop years) were conducted in Federer’s augmented block design (Federer, 1956), with randomized controls in all experimental blocks. Each experimental plot of each evaluated genotype consisted of a 5-m-long row, with a 0.5-m spacing between rows, and a sowing density of 15 seeds per linear meter. The agronomic characteristics were evaluated on five individual plants within each experimental plot, in all populations and in all agricultural years, making up a robust set of evaluated data.

### Discriminant Analyses

Fisher and Anderson discriminant analyses are linear combinations of the observed characteristics that present the best discrimination power among all possible linear combinations of the same characteristics (Johnson and Wichern, 2002). In these methodologies, the total dataset is divided into a training set (80% of the data) responsible for obtaining the discriminant functions and a validation or test set (20% of the data) responsible for validating the functions. For the test set to be a representative sample of the training set, several data partitions were performed, where the mean and variance of each generated set pair were compared, and the pair in which the estimates were the closest possible was selected. Cross-validation of the data and Fisher’s linear and Anderson’s quadratic discriminant functions were performed in Genes Computer Application (Cruz, 2008) according to the methodology of Cruz et al. (2014).

### Analysis by ANN

After several experiments on the best architecture for the multilayer perceptron network type, neural network architecture 12–64–128-11 built in Python 3.6 using Keras as the frontend, TensorFlow 2.3.0 as the backend, and Scikit-learn 0.22.2 was adopted. It was necessary to convert the categorical variable (year) to one-hot representation. Thus, the number of input neurons was 12, corresponding to the POP, NDF, NDM, CYCLE, AIV, APM, Ac, VA, and PG populations and three agricultural years 2017/2018, 2018/2019, and 2019/2020. In the output layer, the number of neurons corresponded to the number of defined classes, that is, 11, and the hidden layers had 64 and 128 neurons, respectively. The dataset had 7,287 examples. The algorithm used to train ANN was stochastic backpropagation (stochastic gradient descent). Adam optimizer was used. The number of training cycles was set as 600 epochs to prevent training from becoming excessive, which could lead to loss of generalization power. The ANN architecture evaluation was based on the evaluation metrics for classifiers that are mostly derived from the confusion matrix generated by the Scikit-learn package. The matrix was obtained from the test data and used to analyze the quality of predictions of the models. The other metrics used were accuracy (hit rates for positive and negative examples), precision (hit rate for positive examples), recall (coverage of correct positive examples), and F1-score (balance between precision and recall metrics). Two procedures were implemented to validate the ANN architecture. The first was the hold-out procedure, which divided the dataset into two random bases: one for the training set with 80% of the data, and the other for testing with 20% of the data. The second procedure was the *k*-fold cross-validation, which divided the dataset into *k* partitions, where *k*−1 were the data for training and k was the set used for model testing. Thus, *k*-models were created, where the data for training and testing were changed for each iteration (Shalev-Shwartz and Ben-David, 2014). The final model evaluation was the average of the metrics of the k models. This procedure is frequently used to validate models with relatively small datasets. *k* was selected as 10. For the activation of neurons, after evaluating other options, the sigmoid–logistic function was used in the hidden layers and the softmax function was applied in the output layer. The best network architecture was established based on the average accuracy, considering the evaluated possibilities, calculated by multiplying the number of neurons in each layer and the possible activation functions. Thus, the most efficient network was chosen for each strategy, adopting the lowest apparent error rate (AER) as a criterion.

## Results

### AER and Model Evaluation Metrics

Table 2 presents the classifications of the evaluated soybean genotypes based on the 11 populations considered in the analyses conducted by the Fisher and Anderson discriminant analyses, in addition to the ANN hold-out and *k*-fold approaches. According to Fisher’s analysis, of the total of 1,517 classifications, a total of 889 were considered as erroneous classifications, which makes an AER of 58.6%. For Anderson’s methodology, in turn, for a total of 1,517 classifications, the number of erroneous classifications was 769, leading to an error rate of 50.59%, which is lower than the AER observed in Fisher. Despite this, both methods presented AER above 50%. In turn, it was observed that the validations of the ANN analyses indicated that of the 1,458 classifications performed, only 119 were considered erroneous for the hold-out approach, leading to an AER of only 8.16%, while, of the 729 classifications for the *k*-fold approach, only 41 were considered erroneous, constituting an even lower AER of only 5.62%.

**Table 2:** Classification of soybean genotypes into 11 populations of different relative maturity groups and estimation of apparent error rate (AER) according to Fisher’s and Anderson’s discriminant analysis and hold-out and *k*-fold ANN approaches.

Approach	Total ratings	Misclassifications	AER (%)
Fisher	1,517	889	58.60
Anderson	1,517	769	50.59
Hold-out	1,458	119	8.16
*k*-fold	729	41	5.62

The superiority of the *k*-fold cross-validation to the hold-out procedure can also be observed from Table 3. This table presents the metrics that assess the quality of the model used in the classification of soybean genotypes belonging to the 11 populations using the *k*-fold and hold-out ANN approaches. *k*-Fold showed accuracy of 93.36%, precision of 93.49%, recall of 93.23%, and *F*1-score of 93.36%, which was greater than the corresponding hold-out scores by at least 1.30% for all metrics. In turn, the highest loss value was presented by the hold-out model (34.10%) when compared to the *k*-fold model (26.39%), in a difference of 7.71% between the two approaches.

**Table 3:** Model prediction quality evaluation metrics for hold-out and *k*-fold approaches in classifying soybean genotypes in 11 populations from different relative maturity groups.

Approach	Loss (%)	Accuracy (%)	Precision (%)	Recall (%)	*f*1-score (%)
Hold-out	34.10	91.84	92.14	91.70	91.94
*k*-fold	26.39	93.36	93.49	93.23	93.36

### Confusion Matrices

Considering the best parametric and nonparametric approaches, the confusion matrices generated from the validation dataset based on the classification by the Anderson’s discriminant analysis and the *k*-fold cross-validation, are presented in Tables 4 and 5, respectively. To interpret a classification within a confusion matrix, the column population is that to which the genotype belongs and the row population is that allocated by the model. Therefore, the correct classifications are on the highlighted diagonal and the incorrect ones outside of it.

**Table 4:** Classification of soybean genotypes in 11 populations from different relative maturity groups according to Anderson’s discriminant analysis.

POP	Brazil	Southern	Northern	GBN1	GB2	GS1	GS2	GN2	TGM7	TGM6	TGM8
**Brazil**	**270**	96	84	2	10	70	7	15	27	28	25
**Southern**	22	**160**	0	0	19	197	3	0	0	40	12
**Northern**	0	0	**174**	3	0	0	0	15	1	0	0
**GBN1**	0	0	0	**27**	0	0	0	0	0	0	0
**GB2**	0	2	0	0	**19**	0	12	0	0	2	0
**GS1**	0	1	0	0	0	**16**	0	0	0	2	1
**GS2**	0	1	0	0	1	0	**17**	0	0	0	0
**GN2**	0	0	1	0	0	0	0	**10**	0	0	1
**TGM7**	0	0	1	0	0	0	0	3	**20**	0	7
**TGM6**	1	23	0	0	1	18	1	0	0	**14**	1
**TGM8**	0	0	0	0	0	1	0	0	8	4	**21**

The column population is that to which the genotype belongs and the row population is that allocated by the model. Therefore, the correct classifications are on the highlighted diagonal and the incorrect ones outside of it.

**Table 5:** Classification of soybean genotypes in 11 populations of different relative maturity groups by *k*-fold approach.

POP	Brazil	Southern	Northern	GBN1	GB2	GS1	GS2	GN2	TGM7	TGM6	TGM8
**Brazil**	**314**	11	0	0	0	0	0	0	0	3	0
**Southern**	4	**205**	0	0	1	4	0	0	0	3	0
**Northern**	1	0	**72**	0	0	0	0	1	0	0	0
**GBN1**	0	0	0	**12**	0	0	0	0	0	0	0
**GB2**	0	2	0	0	**18**	0	0	0	0	0	0
**GS1**	0	2	0	0	0	**11**	0	0	0	1	0
**GS2**	0	0	0	0	0	0	**9**	0	0	0	0
**GN2**	0	0	0	0	0	0	0	**2**	0	0	0
**TGM7**	1	0	0	0	0	0	0	0	**13**	0	0
**TGM6**	3	4	0	0	0	0	0	0	0	**25**	0
**TGM8**	0	0	0	0	0	0	0	0	0	0	**7**

The column population is that to which the genotype belongs and the row population is that allocated by the model. Therefore, the correct classifications are on the highlighted diagonal and the incorrect ones outside of it.

Table 4 shows that the allocation of the genotypes from the GS1 population in the Southern population has the largest error. The Brazil population was the only one to receive incorrect classifications from all other ten populations. Of the 23 incorrectly classified plants belonging to the Brazil population, 22 were from the Southern population. Approximately 34.5% of the plants from the Southern population and 32.6% from the population were classified as being from the Brazil population. There were no misclassifications between the Southern and northern populations.

From Table 5, a large reduction in the number of misclassifications can be observed. The misclassifications that occurred from the genotypes belonging to the Southern population being allocated to the Brazil population contributed most of the 5.62% of errors in the *k*-fold cross-validation approach. The reciprocal case of the genotypes belonging to the Brazil population being allocated to the southern population was also highlighted by the presence of errors. In addition to these pairs of populations, classification errors occurred between the Brazil and southern; GS1 and southern; southern, TGM6 and the reciprocal; and TGM6, Brazil and the reciprocal populations. The GBN1 population did not receive erroneous allocations from any other population and its genotypes were not classified as belonging to other populations.

In addition to the reduction in Anderson’s errors for the *k*-fold cross-validation, differences in the classifications by the two procedures were observed. In the latter, in addition to the GBN1 population, the GS2, GN2, and TGM8 populations did not receive any incorrect genotypes. Moreover, the genotypes of the Northern, GS2, TGM7, and TGM8 populations were correctly classified.

### Annual Evaluation graphic

**Figure 1:**
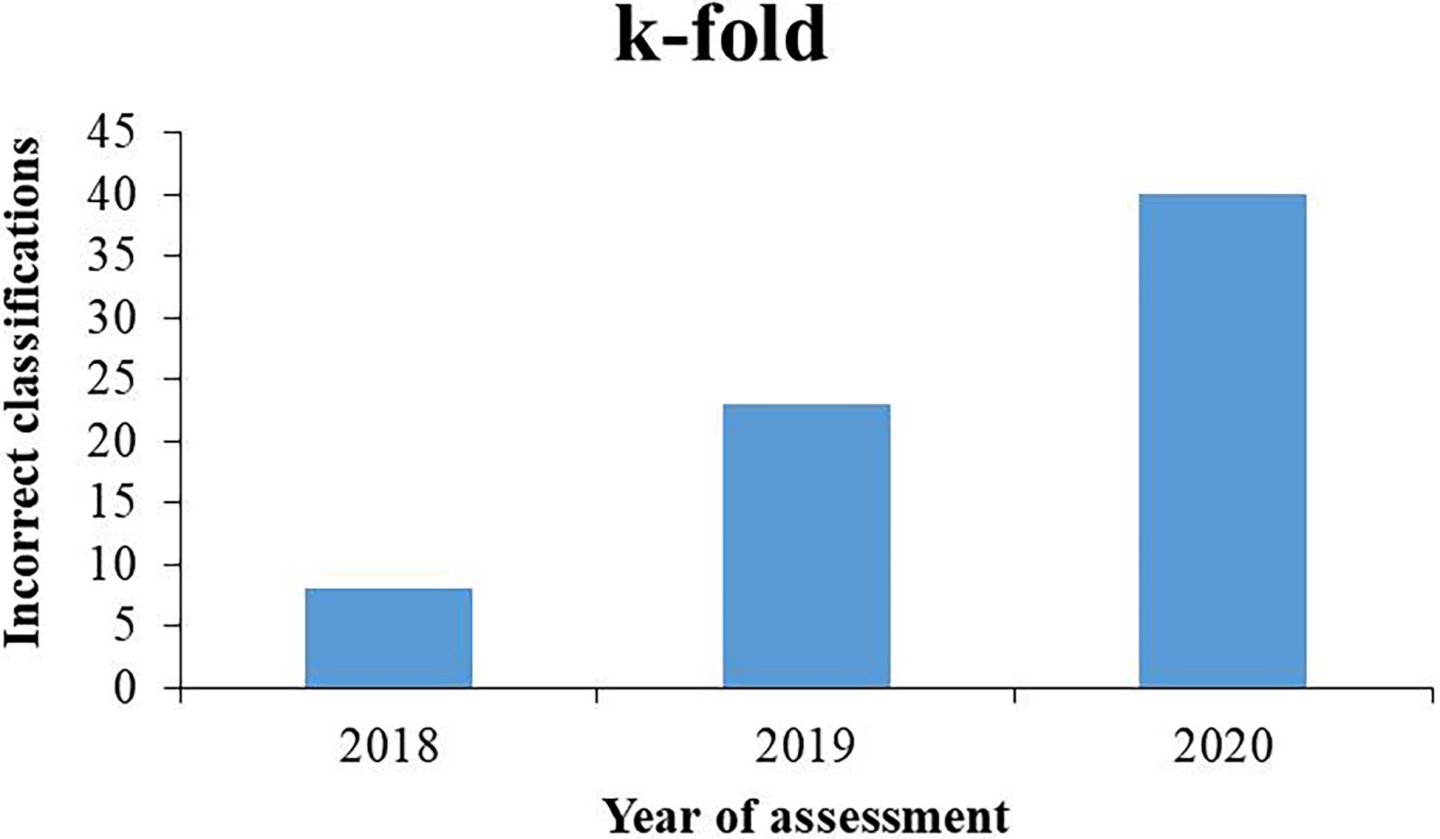
Incorrect classifications of soybean genotypes from 11 populations by k-fold methodology in agricultural years of evaluation 2017/2018, 2018/2019, and 2019/2020.

Figure 1 shows the values of incorrect classifications that occurred in the agricultural years of evaluation of the genotypes 2017/2018, 2018/2019, and 2019/2020 using the *k*-fold approach. From the increase in the bars of the graph that indicate the number of incorrect classifications, an increase in classification errors can be observed from the year 2018 to 2019 and 2019 to 2020, reaching a total increase of approximately 40%, considering the interval between the first and last years of assessment.

## Discussion

The failure to discriminate between genotypes and classify them into their populations correctly is caused by four main factors. First, the populations can be very similar in their origin and genealogy. Second, the number of evaluated variables may be insufficient, in addition to having low discriminatory quality, as a third factor. The fourth cause is the use of an inadequate statistical approach (Cruz et al., 2014). In quadratic discriminant functions such as Anderson’s, with an increase in the heterogeneity of variance and covariance matrices, the nonlinearity of the classification thresholds increases, enhancing the performance in modeling the structure of the function (Carvalho, 2019). The results observed in the present study corroborate with Carvalho (2019), considering that Anderson’s methodology was 8.01% more accurate than Fisher’s methodology. However, both discriminant functions consider parameters and assumptions that are frequently insufficient for explaining a dataset. The occurrence of the AER above 50% of the examples suggests the limitation of these methodologies in discriminating the genotypes in this study, particularly when considering the population genetic structure and their inefficiency in classifying the genotypes. In contrast, ANNs learn with experience by exploring the features contained in the data, which is capable of increasing the accuracy of the information obtained in a more detailed manner (Silva, 2019). This study proves that *k*-fold cross-validation is considered an accurate and suitable approach for small datasets (Shalev-Shwartz and Ben-David, 2014). The evaluation metrics of the models’ accuracy, precision, recall, and F1-score point to the higher quality of the models as their values are closer to 100%. In turn, the loss points to a higher quality depending on how low is their presented value. The efficiency of the *k*-fold approach in correctly classifying more than 94% of the examples shows its superiority to the hold-out procedure. Moreover, the former has higher accuracy (93.36%), precision (93.49%), recall (93.23%), and f1-score (93.36%) than the latter. The higher loss presented by the hold-out approach (34.10%) compared to *k*-fold (26.39%) confirms the latter’s higher classification efficiency. In terms of percentage, the less efficient ANN validation approach correctly classified 40% more genotypes than the more efficient discriminant function.

The proximity of the origin and genealogy of genotypes belonging to different populations largely contributes to the inefficiency of their discrimination, as also observed by Sant’anna (2014) in the study on ANN and backcross populations with different degrees of similarity. Anderson’s discriminant function was inefficient in discriminating genitors from their descendants because most of the incorrect classifications that occurred with GS1 genotypes, represented by the BMX Potência RR cultivar, were allocated to the southern population, which is its descendant.

The Brazil population presents a high level of genetic variability in the relative maturity, resulting in the presence of many allelic combinations of genes that control the timing of flowering and maturity (Lin et al., 2021). This was also a limiting factor for the good performance of the parametric methodology, causing it to classify genotypes from all other populations as belonging to the Brazil population, equivalent to 24% of the total errors. This capitalizes all variations in populations with narrow genetic basis and cultivars of pure lineages. Despite this, in the Brazil population, only 23 plants were misclassified, that is, 7.8%, and 22 of these were allocated to the Southern population. This shows that it is broadly representative phenotypically of all other populations, even with little similarity to the Southern population, characterizing the wide genetic variability in the Brazil population. In contrast, 34.5% of the plants in the Southern population were classified as being from the Brazil population, which does not present a relationship or known biological explanation. Thus, the applied statistical technique is the probable cause of the incorrect classifications (Cruz et al., 2014). In the Northern population, 32.6% of the individuals were classified into the Brazil population. In this case, the two populations have a common genitor, which may also be a biological cause of the misclassification. Between the Northern and Southern populations, there were no misclassifications, and there were also no relationships between the genitors.

When considering the genitors of the populations and the other three witnesses, it was noted that in the Northern population, fewer misclassifications occurred compared with that in the other populations; however, the errors of classification, in general, were always large, both in absolute values and percentages. There was a tendency for no differentiation of the genitor with the derived population, as in the case of GS1, Brazil and Southern populations and GN2, Brazil and Northern populations. Witnesses, even being pure lines, were erroneously classified as belonging to different populations, mainly for plants of the Southern population in the TGM6 witness. Thus, the statistical technique is inefficient in classifying pure lineage genotypes or those with a narrow genetic base and is partially effective even in populations with a broad genetic base, such as the Brazil population.

The only case in which no error occurred using Anderson’s methodology was the GBN1 population, corresponding to the BRS 278 RR cultivar with RMG 9.4, which did not receive incorrect genotypes from any other population. The RMG of this population, considered late for the evaluation region, induces elongation of the vegetative stage, resulting in extremely tall plants with low grain production, contributing to an atypical phenotype of this cultivar in Jaboticabal, the ideal RMGs for which are between 6 and 8.

Based on the confusion matrix obtained by the *k*-fold cross-validation, the wide variability of the Brazil population also led to misclassifications of the genotypes from the Southern population. However, the number of errors by this method was 11 compared to 96 errors by Anderson’s discriminant analysis for the above pair of populations. The other *k*-fold misclassifications comprising the 5.62% errors occurred between Brazil and southern; GS1 and Southern; southern, TGM6 and the reciprocal; and TGM6, Brazil and the reciprocal populations. However, the number of errors was always equal to or less than 4. The non-occurrence of misclassification errors in the GBN1, GS2, GN2, and TGM8 populations as well as the absence of incorrect allocations in the Northern, GBN1, GS2, TGM7, and TGM8 populations demonstrate that, even with a relationship between the genitor and its derived population, ANN is very efficient for broad-based populations. Furthermore, the ANN correctly classifies the narrow-based populations and pure lines, learning to differentiate genotypes. Although the classification scheme of the ANN was affected by the genotype, it was to a lesser extent than Anderson’s discriminant method.

The mechanical evaluations based on the traditional characteristics in the three considered years at the same location did not impair the performance of the ANN; however, it shows the poor influence of noise and data loss on the quality of the information obtained (Silva, 2019). Furthermore, the correct identification of the genitors and witnesses shows that the genetic proximity between the genotypes and the existing genetic variability, whether wide or restricted, does not limit to the result accuracy. This demonstrates the usefulness of the technique in breeding programs even in the F_6_ and F_7_ generations.

The built ANN identified the genotypes that were incorrectly classified by their corresponding numbers in the data table. From this information, it was possible to identify the generation of self-fertilization of each of these genotypes based on the year of evaluation. As the generations advance in homozygosity and selections are made within the populations, mainly based on productivity, they become uniform and similar to each other because they are generally intended for cultivation where they are located, as also identified by Sant’anna (2014) using simulated data. Genotypes grown outside their ideal photoperiod conditions present reduced productive potential (Lin et al., 2021) and are eliminated in the selection process. The increase in incorrect classifications from 2018 to 2020 reached approximately 40%, which shows the importance of classifying and assigning genotypes to their appropriate evaluation regions. At this stage of the local breeding program, it would be ideal if the advanced lines in F_5_ or F_6_ were evaluated in the target region according to their photoperiod. The dataset also leads to the conclusion that lineage development in an intermediate region (RMG 7) is suitable for advancing the lineages for extreme photoperiods, as for RMGs 5 and 9.

In a conventional soybean breeding program, many resources are used to obtain a successful cultivar. Time and money are invested in researching, testing, selecting, and developing promising genotypes. After the selection stage, it takes at least 5 years for the implementation of tests in the intended cultivation regions for the commercialization of the cultivar and it can be extended up to 10 years until the launch of the final product (commercial cultivar) (Seednews, 2019). This study demonstrated that neural networks are able to classify genotypes and discriminate them for their ideal ranges of cultivation even in early generations, which would lead to a reduction of resources spent between the selection and testing steps in the existing edaphoclimatic macroregions. In addition, the high efficiency of ANNs in discriminating populations with wide and narrow genetic variability allows their application to obtain genotypes to be tested throughout Brazil, from the same base population of wide genetic diversity, even in early and intermediate generations of inbreeding, for the development of new cultivars, enabling the exploration of greater genetic variability from wider crosses and contributing once again to the reduction of time and resources invested in genetic improvement programs.

## Data Availability Statement

The raw data supporting the conclusions of this article will be made available by the authors, without undue reservation.

## Author Contributions

LA: planning, implementation, and experimental conduction, field evaluation, analysis and data collection, and text writing. GM: analysis and data collection, text writing, and correction and guidance. BV: implementation and experimental conduction, and analysis and data collection. AS: implementation and experimental conduction, field evaluation, and analysis and data collection. AM: implementation and experimental conduction, field evaluation, and analysis and data collection. SU-T: planning, analysis and data collection, text writing; correction and guidance, and work leader. All authors contributed to the article and approved the submitted version.

## Funding

This work was supported by the Pro-rectory of Postgraduation (PROPG), São Paulo State University - UNESP/FCAV Jaboticabal.

## Conflict of Interest

The authors declare that the research was conducted in the absence of any commercial or financial relationships that could be construed as a potential conflict of interest.

## Publisher’s Note

All claims expressed in this article are solely those of the authors and do not necessarily represent those of their affiliated organizations, or those of the publisher, the editors and the reviewers. Any product that may be evaluated in this article, or claim that may be made by its manufacturer, is not guaranteed or endorsed by the publisher.

## Supplementary Material

Supplementary Table Dataset collected from soybean progenies belonging to different relative maturity groups, during three agricultural years of evaluation.
